# The Extended Congress: Reimagining scientific meetings after the COVID-19 pandemic

**DOI:** 10.15694/mep.2020.000128.1

**Published:** 2020-06-22

**Authors:** Alvaro Margolis, Jann Torrance Balmer, Andrea Zimmerman, Antonio López-Arredondo

**Affiliations:** 1 EviMed; 2 University of Virginia; 3 EviMed; School of Engineering

**Keywords:** Internet, Learning Analytics, Continuing Medical Education, elearning, learning technologies, medical conferences, scientific meetings, change, COVID-19, crisis management

## Abstract

This article was migrated. The article was marked as recommended.

The COVID-19 pandemic has profoundly impacted the medical meetings planned for 2020. This health crisis has caused the cancellation, postponement or a pivot in educational design to virtual formats. In the latter case, the format for virtual meetings has remained very similar to the cancelled face-to-face meeting, by using primarily web conferencing systems.

This article intends to start a dialogue with the medical education and events community about possible delivery formats. Among them, the concept of an “Extended Congress” is introduced.

The extended congress uses the extension of time, space and languages to a scientific meeting. It aims to: 1) unleash the reach of traditional meetings through the use of technology to access larger audiences in different languages, across a country and internationally, with local leaders to help interpret the knowledge and localize it, and 2) to improve knowledge translation into practice through a sequential and active learning process.

An ongoing example is described as a proof of concept: the Latin American Peritoneal Dialysis Extended Congress attracted 774 remote participants from over 20 countries, 93% of whom were paid registrants. Initially designed as a hybrid (live plus remote) event scheduled for March 2020, it had to be reframed as a remote only meeting due to the COVID-19 pandemic, thus protecting the health of members while providing continued value to the organization and attendees of the event.

With this experience in mind, the authors are currently designing programs in the United States, through collaboration with the University of Virginia Office of Continuing Medical Education.

In summary, the design of meetings can better utilize and integrate technology and reach larger audiences with a blend of formats. Those organizations that adapt more quickly to offer these events will concentrate more of the share, as seen with the adoption of technology by other industries.

## Introduction

The COVID-19 pandemic, among its many effects in almost every facet of our lives, has profoundly impacted the medical meetings planned for 2020. For the meetings and conferences scheduled in the first half of the year, organizations were faced with decisions such as cancellation, postponement or a pivot to virtual formats ( McKimm *et al*., 2020; McGowan, 2020; Gravitz and Frellick, 2020), with little time for planning. In the latter case, the virtual formats selected, due to the speedy implementation, have remained similar to the structure of the cancelled face-to-face meetings, using primarily web conferencing systems such as Zoom, or sometimes recorded lectures, with little interaction among participants, mostly through open social networking applications such as Twitter, LinkedIn and Facebook.

In many occasions, these scientific meetings that were modified to virtual formats were provided at no cost, as shown in this example ( ACC, 2020), which added to the negative financial impact of the organizations involved, already strained because of the cancellation of the live event, with new obligations and less income.

Furthermore, this traditional format - with live activities via web conference - is not well suited for the remote participant, because it is not feasible to keep the participants’ undivided attention through internet-based activities over many hours and days, and often in different time zones. In a live meeting, almost all of the participants’ time is devoted to the meeting, but in a virtual event their dedication is highly impacted by their daily work obligations and family life, therefore requiring a more realistic and staged approach that allows for flexibility in participation.

For the meetings scheduled for the second half of 2020, a decision to convert to a virtual format allows more time for planning and implementation to provide a better educational and networking design ( AMEE, 2020). As organizations plan to review their schedule for live meetings and conferences with a traditional format, there are still options to incorporate technology and extended participant engagement. Regardless of the familiarity and value proposition of a live face to face meeting, planners need to actively explore contingency plans for the increased use of technologies to augment or replace the essential content being delivered to participants as well as social interaction among attendees. As we plan for 2021 and beyond, the changes in medical meetings and live activities need to consider multiple approaches that include technology.

Please note that the term “medical meetings” is used in this article to refer to scientific conferences for healthcare practitioners and those in related fields. Moreover, many of these concepts could be applied to other industries where scientific meetings take place.

This article intends to start a dialogue with the medical education and events community about possible delivery formats, that may be helpful to the medical organizations and meeting organizers in charge of coming events, along with other colleagues who are reflecting on these topics and having an open dialogue with the medical education community through this Journal ( Sandars *et al.,* 2020; Fawns, Jones and Aitken, 2020). Among possible delivery formats supported by information and communication technologies, the concept of an “Extended Congress” is introduced as an alternative.

## The Extended Congress

### Definition

The concept of “ **Extended Congress**” ( Margolis, 2020) leverages the extension of time, space and languages of a scientific meeting into a framework where learning can be integrated with the demands of work/life. For example, a large conference in Infectious diseases or in Cardiology in the US or Europe, or a smaller medical meeting as well, could be extended in *space* (to reach a larger audience who would not normally participate in that conference). This increased accessibility could include domestic and foreign live and remote participants from different states, countries or continents, allowing participants to be able to both attend and present their research live or remotely. Expand *time* by extending the activity over 4 to 8 weeks, to allow those live attendees and those who attend remotely, to network with each other and to acquire the valuable new knowledge and validate it with colleagues through an engaging conversation ( Margolis *et al.,* 2019). Importantly, this Extended Congress model also fosters delivery in *languages* (allowing to those who do not master English - or another language that is the official language of the event - to participate, with subtitled lectures and discussion forums in their own languages).

Furthermore, distributed live activities in English and/or local languages during the congress could coexist, at different locations of the countries or the world, either face-to-face or remotely through the Internet, led by local congress ambassadors, taking into account the possible decreased international mobility of participants after the pandemic, and willingness or not of participants and speakers to attend crowded spaces. Finally, a possible spin-off of the live meeting could be the Conference highlights, delivered later in time, summarizing and discussing in English and/or local languages the key results of the meeting over a period of 6-8 weeks, similar to a massive open online course (MOOC), thus evolving from its traditional format (ASN, 2019).

Although this concept was originally designed to be incorporated into hybrid events with a central live meeting as an integral part of the educational plan, the conversion to a fully virtual Extended Congress approach could serve as a substitute for the live or hybrid event, because of the travel and gathering restrictions that are currently in place for this year. In any case, the *virtual extended meetings* could become a new type of event with its own merits for these organizations, together with the hybrid and traditional live meetings.

### Rationale

Professional communities of specialists benefit from meetings as a dedicated space for sharing the latest research, and interacting with peers and experts. Many reputed conferences already host a large international audience, and the associations that organize them have year-long relationships with national and regional societies of the same specialty (ESC, 2020; ACC, 2020). However, unleashing the reach of these events through the use of technology could eventually help access even larger audiences, across a country and internationally. If language barriers are minimized, the impact of the education and research can be expanded. Finally, a better educational design, with group discussions leading to collective reflection, and sequential activities over time, could help knowledge translation into practice, a feature that traditional congresses frequently lack ( Davis *et al.,* 1999; Mansouri and Lockyer, 2007).

### Prior experiences

The concept of “virtual congress” is not new. In fact virtual congress approaches have been around for over two decades ( Lecueder and Manyari, 2000). A number of scientific societies have introduced technology into their meetings in various ways, such as the use of smartphone Apps and social networking applications (e.g. Twitter) during the conference, conference summaries through Webinars or recorded videos, or availability of congress sessions as enduring materials. While these approaches improve and enhance the learning, they are not intended as a comprehensive solution for those who cannot attend onsite or designed to create an engaging learning experience over time for those who did attend.

Moreover, the concept of congress highlights available months after a conference are a well-recognized feature of some major international conferences, such as the ASN highlights ( ASN, 2020). This distribution approach helps to provide information but these events are often delivered onsite at different locations over one day, often in English. In considering technologic enhancements, the delivery of primarily didactic content can be re-envisioned to foster greater engagement and application of new knowledge. Also, two of the authors’ team (AM/AL) have experience in creating and delivering geographically distributed live face-to-face or remote activities as launching events of international online courses ( Cohen *et al.,* 2014). The conceptualization of a multi-modal educational design can foster the integration of remote presentations of research and exchange during the live meeting, as in the original design of the AMEE 2020 live conference and also in its current virtual format.

However, none of these examples mentioned above comprehensively address the needs of the different stakeholders involved in a medical meeting, particularly the potential *participants* in the healthcare community (attendees and potential attendees, researchers, speakers) and the *institution* organizing the meeting, as well as the commercial supporters, the professional conference organizers, and other vendors and contractors involved in the conference services industry.

Meetings serve as a primary source of value, revenue and networking for its members and attendees. The customs and patterns of engagement associated with traditional live meetings help organizations to share their organizational identity and have been a consistent source of revenue. Major changes that impact the organization’s value proposition can be viewed with suspicion and anxiety. The consideration of a new approach is now a reality due to the pandemic and requires a re-assessment of existing practices as these decisions may ultimately affect overall sustainability for the organization, and learning experiences, after the assessment of ongoing implementations, will be available.

## A proof of concept: the Latin American Peritoneal Dialysis Extended Congress

Two of the authors’ team (AM/AL) envisioned the concept of technology-based education through Extended Congresses for years, but the risk benefit equation was not reasonable for many associations and organizations. However, in October 2019, the Colombian Association of Nephrology, organizers of the 7th Latin American conference of Peritoneal Dialysis, accepted the challenge and the project was launched (Extended Congress, 2020).

**Figure 1.**
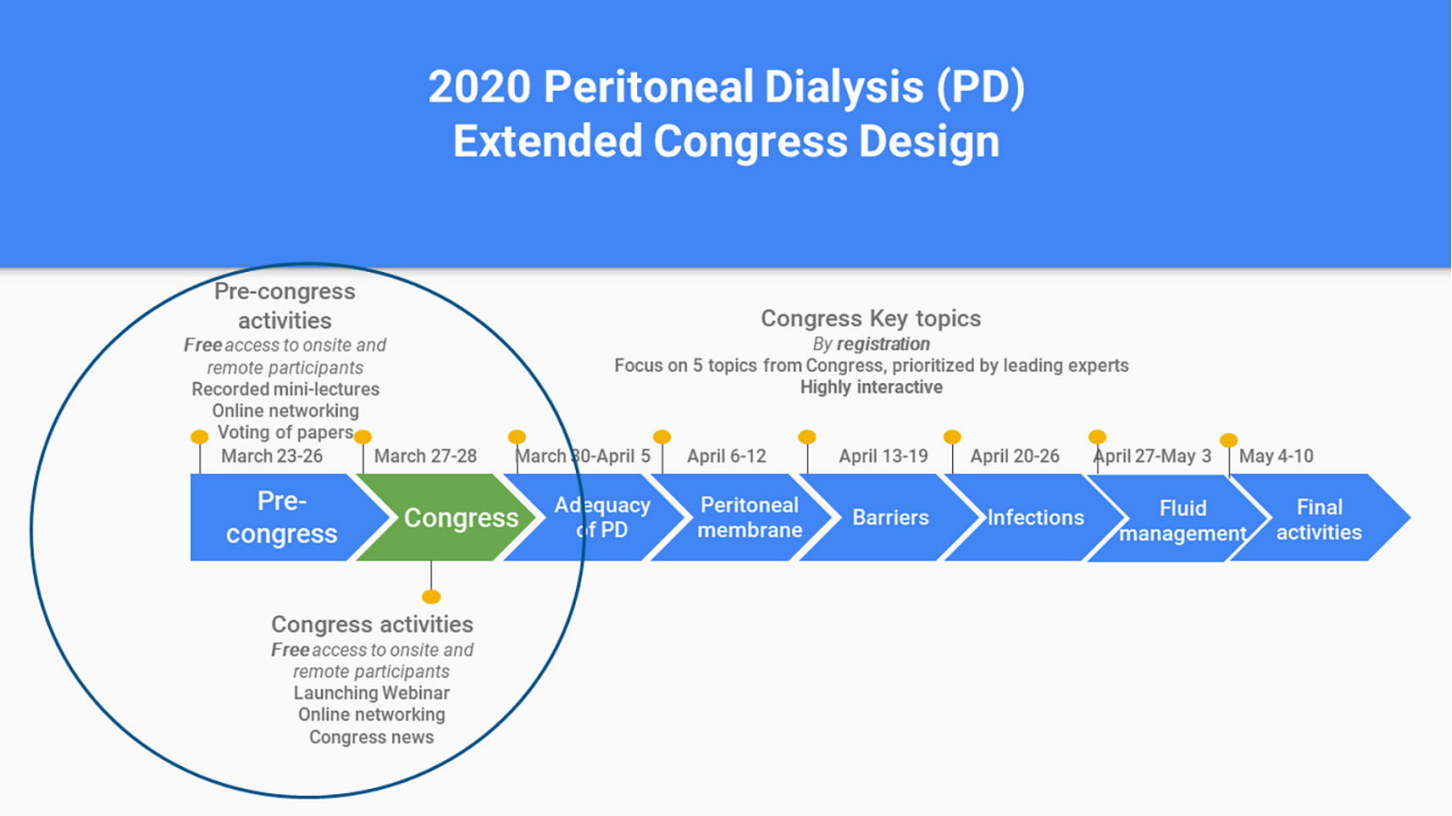
Peritoneal Dialysis extended congress design

The design of the Peritoneal Dialysis Extended Congress is shown in Figure 1. As it can be seen, it is built on the foundation of a live two-day conference, which also included as parallel sessions other topics besides peritoneal dialysis, in partnership with Mayo Clinic. The live conference was scheduled for March 27-28, 2020 and was focused on Colombian healthcare professionals and those from neighboring countries (around 300 to 400 attendees). The addition of the extended congress created an opportunity to enhance wider reach. This activity utilized a platform familiar to a number of physicians and healthcare professionals who usually take online courses in nephrology in the region and was endorsed by the Latin American Society of Nephrology (SLANH), a trusted partner for the attendees, as well as the International Society of Peritoneal Dialysis (ISPD).

**Figure 2.**
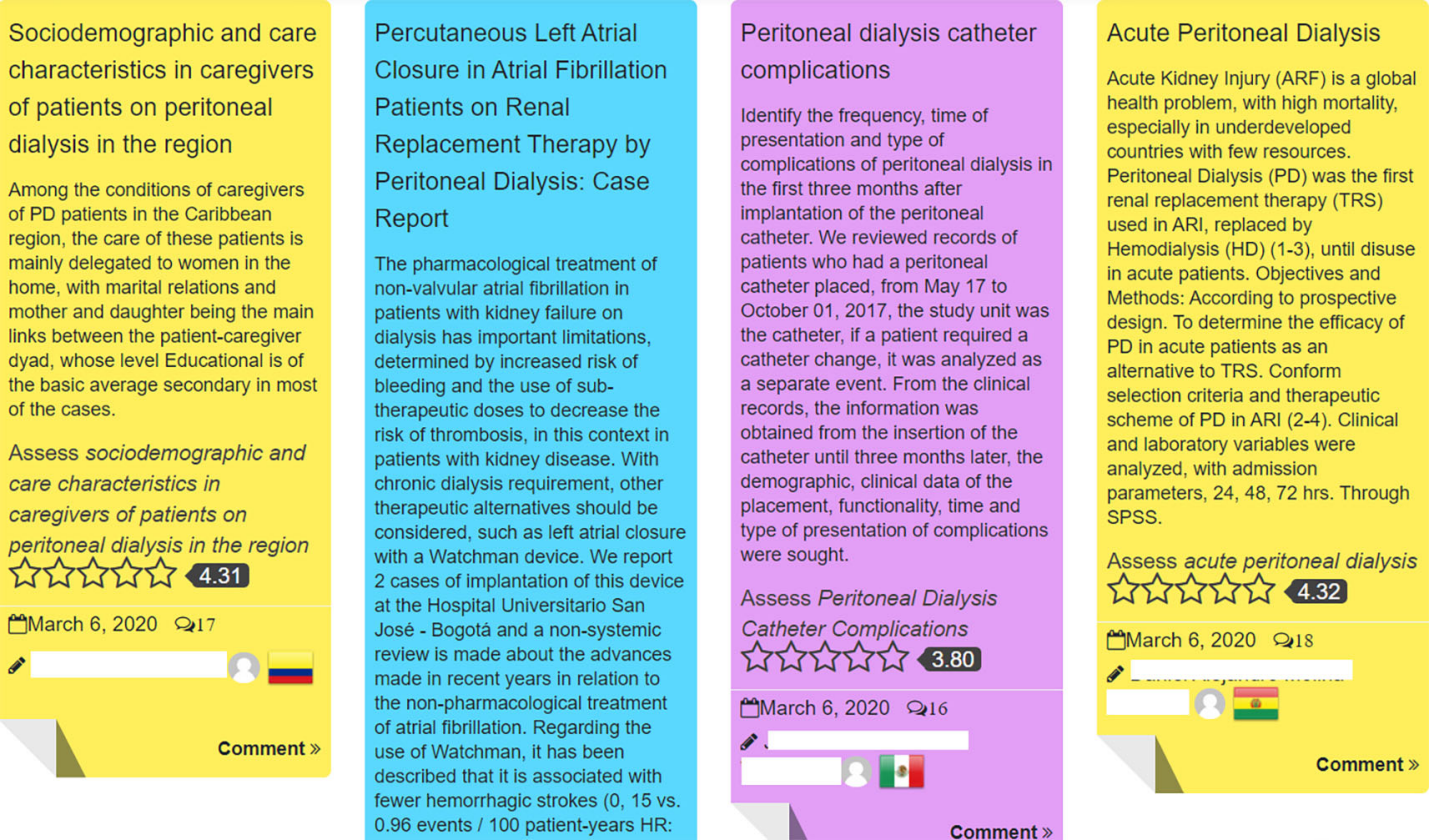
Sample of papers submitted. Interactions by participants with researchers, by voting and by comments

The design of the extended congress included:

- The first week, when the live conference takes place (“pre-congress” and “congress”, March 23-28, in Figure 1), provided both onsite and remote participants with full access to the virtual component of the congress, and those remote participants who started the registration process but did not finish it had free access to this week as well. This week included aspace for participating in online networking, recorded mini-lectures, and a review of papers and posters accepted to the conference, where participants could vote and interact with researchers (see Figure 2). Also, congress news were planned to be written by local correspondents at the conference. A launching Webinar of the Extended Congress, summarizing the main outcomes of the event, was planned.

- The following five weeks after the initial conference session (weeks of March 30 to May 3), were focused on the main topics of the conference, addressed with short lectures, reading materials, clinical simulations and case discussions in clinical forums. Collective reflection with peers and experts is maximized by interaction and participation with experts and tutors and the use of social networking tools in the platform ( Margolis *et al.,* 2019). This part of the extended congress required a registration fee.

- A final week (May 4-10), included closing discussions and a farewell forum.

In addition, a nursing track was specifically designed for the extended congress, thus allowing an important segment of the target audience related to peritoneal dialysis to have a unique set of content and activities, tutored by nephrology nurses.

### Pivoting to virtual

By March 11, 2020, less than three weeks before the scheduled date for the live congress, the face-to-face event was cancelled because of the COVID-19 pandemic. In light of this change, all participants were encouraged to participate in the virtual component of the congress where most of the characteristics of the live event were included, particularly the presentations of research already accepted and coverage of the main topics of the congress. Naturally, the conversations and discussions include the use of peritoneal dialysis and COVID-19. The extended congress is now progressing, including discussions about the higher value of peritoneal dialysis in an era of confinement, or the use of peritoneal dialysis in COVID-19 related acute kidney injury.

### Country distribution of participants

As of April 10, the distribution of participants to the extended congress is similar to anticipated expectations. The extended congress has a wide attendance from over 20 Spanish-speaking countries of the region and Brazil, Spain, USA and Portugal ( Table 1).

**Table 1.** Distribution of participants by country

Country	Participants	Percentage
Mexico	160	20.7%
Argentina	131	16.9%
Costa Rica	94	12.1%
Peru	85	11.0%
Chile	57	7.4%
Colombia	48	6.2%
Ecuador	41	5.3%
Uruguay	35	4.5%
Venezuela	21	2.7%
Brazil	20	2.6%
El Salvador	18	2.3%
Guatemala	16	2.1%
Bolivia	14	1.8%
Portugal	5	0.6%
Honduras	5	0.6%
Paraguay	5	0.6%
United States	4	0.5%
Panama	3	0.4%
Spain	3	0.4%
Dominican Republic	3	0.4%
Other	6	0.8%
**TOTAL**	**774**	**100.0%**

Seven hundred and twenty (93%) of the attendees are paid participants; the other 54 (7%) are full scholarship participants from Venezuela and others who requested them. Additionally, another 1986 professionals were granted free access to the initial week, as pre registrants to the activity, which is done automatically with all the educational activities: 393 (20%) of them accessed the platform. A full report of the congress statistics will be available following the conclusion of the congress.

### “Vanilla” version of the extended congress

In software jargon, a “vanilla” version is when the software is not customized, when it is the plain or standard version. When this activity was implemented as a proof of concept, we decided to run it with a minimum viable set of functionalities (just “plain vanilla”, with nothing else added), taken from the online courses that we already were implementing ( Margolis and López-Arredondo, 2019). Therefore, the educational format of each of the weeks, the starting week, the free initial period to all participants (including pre registrants who did not pay), the social engagement strategy ( Margolis *et al.,* 2019) and the launching live event were taken from the online courses and repurposed in meaning for this activity. Still, some new functionalities were added, particularly a feature that allowed the possibility of discussing papers and posters accepted to the congress on the platform, which has been found to be quite engaging ( Figure 2).

**Figure 3.**
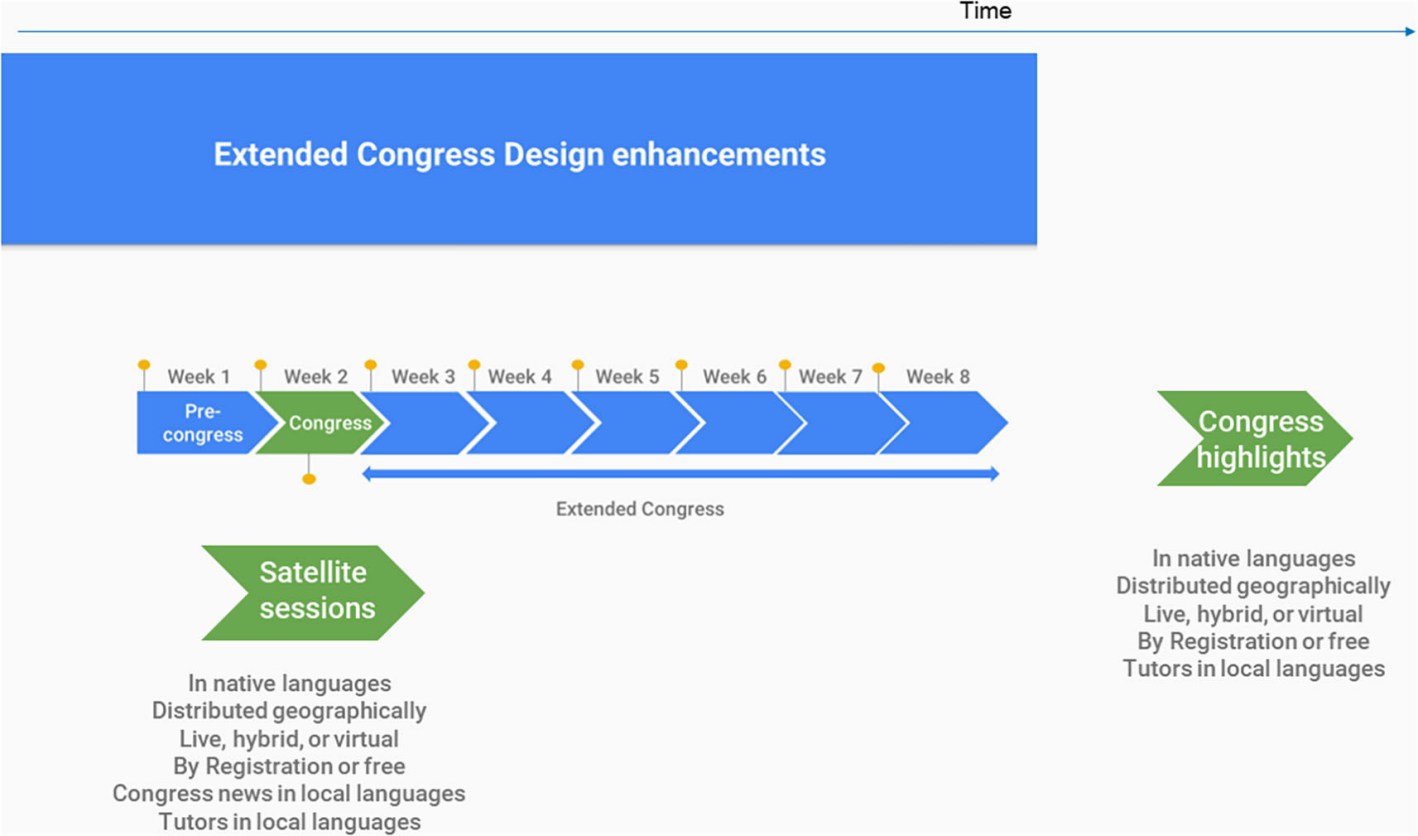
A more developed model of the extended congress

The decision of focusing on Spanish-speaking participants mostly (and not Portuguese-speaking ones) was selected, to minimize risk. However, faculty presenters were diverse, speaking English, Portuguese and Spanish, with lectures being subtitled and slides translated into Spanish when necessary. More functionalities and a more comprehensive model could be implemented in following programs, after a formal assessment of this pilot project ( Figure 3).

### Challenges faced when implementing a hybrid event

The concept of extended congress in normal times is received with anxiety by the conference organizers, because of the perceived risk of potential damage to the onsite attendance. In this example, the authors saw an opportunity to implement it, because the local attendees were already subsidized by the national association to participate onsite, and not too many international attendees were expected; at the same time, the conference organizers were keen to implement innovative formats, based on recent experiences together.

A second challenge, particularly when the event is fully virtual, is to implement a reasonable alternative for the live exhibit hall, because the budget for traditional events usually is substantially supported by exhibitors. In our example, there was no commercial funding for the virtual component (income was based on registration fees), but this is not generally the case.

A third potential challenge is to enroll remote participants in these activities: in this example there was a good reach, but it is something to consider.

Finally, ensuring online networking is paramount, as in any congress.

## Programs being implemented by the University of Virginia School of Medicine and Nursing

The University of Virginia (UVA) Office of Continuing Medical Education - CME - (UVA, 2020) has been involved in web-based and innovative continuing education for physicians, nurses and healthcare professionals for over twenty years. The expansion of hybrid continuing professional activities to integrate live and pre-produced content (lectures, posters, case presentations) creates new opportunities for incremental learning and professional development. Educational programming, through the extended congress or other innovative designs, can create a nimble and responsive environment that is accessible to healthcare professionals and teams as needed. The University of Virginia is actively working to utilize platforms such as the extended congress approach, to enhance networking, active learning that is relevant, timely and evidence-based. The COVID crisis has significantly changed the accessibility of continuing professional development and creates an urgent need for asynchronous learning opportunities that can be utilized without leaving the work environment. We are designing courses and activities that can benefit multiple target audiences, consistent with the changing care environments, team member roles and responsibilities and need for comprehensive quality care. The extended congress is particularly helpful as we can modify the balance of live and hybrid content as needed to meet the needs of our target audiences and stakeholders.

The University of Virginia Office of CME and SONCE (School of Nursing continuing education) are working collaboratively to create programming that leverages the expertise of our faculty, our professional joint providership relationships and to drive substantive discussions based on current research and clinical evidence. The University of Virginia collaborated with EviMed, Hospital Italiano de Buenos Aires (a main academic center in Argentina) and the Global Alliance for Medical Education - GAME- on a regional meeting in Latin America focused on advances in continuing medical education in 2015. This initial collaboration fostered new ideas and approaches that are now being integrated into much of UVA’s continuing education (CE) and Performance Improvement opportunities.

UVA is now exploring how to pivot much of the programming into hybrid formats. The need for virtual learning is now a critical component in quality CE as we are aware that healthcare institutional resources are going to be limited. In spite of these financial challenges, there has never been a greater need for highly relevant and timely CE programming. Everyone in the healthcare team can benefit from quality CE that is workplace based and relevant to the provision of quality patient care. The changes in the delivery of healthcare that utilizes telehealth technologies also increases the familiarity and ease of technology-based solutions. UVA, like many academic institutions, includes regularly scheduled series (RSS), departmental conferences as well as asynchronous web-based continuing education. Our joint provider organizations need our guidance and leadership to create ongoing CE opportunities for their constituent audiences.

The transition of our CE programming, particularly in light of the changing healthcare environment, is moving at a rapid pace. We are actively creating alternative approaches for our course directors and planning committees that reflect this new reality. We plan to leverage our long-standing experience in web-based education into a broad-based armamentarium of educational design options that can be tailored to the needs of our learners. The process we use is shown in “Supplementary File 1”.

## Other models: Is virtual reality the solution?

When going virtual, an appealing solution could be to use Virtual Reality (VR), as several organizations have ( Fink, 2020; Begg, 2020). According to the Oxford Dictionary, Virtual Reality is “the computer-generated simulation of a three-dimensional image or environment that can be interacted with in a seemingly real or physical way by a person using special electronic equipment, such as a helmet with a screen inside or gloves fitted with sensors.” There is extensive and successful experience in the use of virtual reality in medical education ( Walsh *et al*., 2020; Heather, Chinnah and Devaraj, 2019).

In practice, two ways for the use of VR in medical meetings can be envisioned:

• *Without special VR lens*: VR is an added presentation layer, similar to the way Street View is an added layer to Google Maps. It looks like a computer game. The question is how much this additional layer adds to the main layer (i.e., lectures, panels, masterclass sessions, research paper and poster presentations, polls, chats and other types of interactions), where the main value to the participants lies. Additionally, how much of a barrier will it become, in terms of complexity and development cost, hardware and bandwidth, participant’s technological ability, among other concerns. There might be niche uses in specific parts of the virtual meetings, for example by integrating into gamification.
• *With special VR lens*: It is a very immersive experience, like a live conference. The problem is that with current technology VR with lens is not tolerable for long periods of time, in contrast to a live conference where participants often spend all day over several days engaged. Additionally, its use adds hardware requirements (the VR lens) that are not yet of general usage. There might be niche uses in specific parts of the virtual meetings, for example in skills-based simulation workshops.

In both cases, the effort to develop these solutions is significant. There certainly will be early adopters of it ( Sahin, 2006), but the authors have doubts about the actual acceptance of this technology as the comprehensive solution for a virtual meeting, due to the low added value, therefore with a low return of investment, and the technical complexity of implementing it. As mentioned above, there might be specific sessions of the meetings that could benefit from using VR.

Technology evolves over time, and features may change in a way that will make it easier to adopt in the future. Right now, the added value of Street View to Google Maps is an analogy to keep in mind.

## Discussion

The impact of the COVID-19 pandemic on scientific meetings has been daunting. It is summarized in ( PCMA, 2020): “ *What has been your biggest challenge professionally and at your organization during the pandemic*? The responses fell into these main buckets: dealing with the loss of income or revenue; uncertainty preventing their ability to plan “intelligently”; communication issues; difficulty convincing leadership of the need to pivot to digital; canceling events and managing contracts, damages, and payments; getting a handle on producing a virtual event; managing their teams, particularly during this time of stress and uncertainty; and navigating the possibility of having to change their own roles in the wake of event cancellations.”

This pandemia precipitated the need to integrate technology. The Extended Congress and other virtual conferences have demonstrated in practice that a significant proportion of the human learning and interaction can be implemented online with reasonable results, thus speeding the adoption of digital technologies in various aspects of our lives. Scientific meetings are not the exception, and they could meet their key objectives (knowledge transfer and networking) by introducing new approaches to educational delivery that could increase their reach, knowledge translation into practice, and encourage global discussions and intercommunication between peers and course faculty.

This paper addresses how technology can help address the impact of the pandemic on 2020 medical meetings. In turn, these ongoing experiences will provide evidence to decision-makers that can influence the long-term impact of the introduction of technology to all stakeholders involved. For example, while conference organizers worry that remote activities risk onsite attendance, in our experience this consequence does not happen ( Lombardi *et al.,* 2018; Margolis *et al.,* 2015). On the contrary, in this example the international profile of the regional association is now much higher than a few years ago: therefore their traditional live conferences have a large onsite participation.

Furthermore, as mentioned before, commercial exhibitors are an important component of the budget related to medical meetings. The revenue generated by exhibitors and vendors creates a significant reason to consider creative options when pivoting to virtual ( PCMA, 2020; SACME, 2020). Accreditation standards require that planners should ensure that “commercial support should follow principles of fairness, transparency, and separation of promotion from education” ( International Academy for CPD Accreditation, 2018). The search for creative ways to address their presence under these rules, providing similar value to exhibitors and participants, needs to be tested. As expressed by a participant in ( SACME, 2020), the challenge is “how to recreate the exhibit experience”, connecting with a remote audience (which in turn could be even larger than the onsite face-to-face registrants).

In the coming years, we will see a mix of hybrid events (where the live component may have a large central location and satellite ones) and virtual only events, based on the explosion of experiences during this year, where organizations take more risks than usual (because the usual *status quo* is not possible or even reasonable). As stated by a participant in ( PCMA, 2020), “.. it forced my whole organization to rethink our event design. I didn’t have to sell anyone on a new tool, because everyone is open to tech now.”

The positive elements of this new model are extending the reach of the main events, including different regions of the world and different languages, acting as an insurance policy (both for speakers’ availability and primarily for the event itself), and improving the educational impact on practice. Regarding the insurance aspect of designing a virtual component, it is expressed by a participant in the PCMA survey ( PCMA, 2020), “I’d incorporate a virtual component from the beginning. Don’t play catch up - refocus energy to something you’ve already built rather than scramble to reinvent the wheel.”

Finally, a concentration effect with the introduction of digital technologies can be expected for the major conferences of each specialty, as shown with the entertainment and news industries ( Elberse, 2013; Lee and Molla, 2018). Quoting the conclusions reached by Harvard researcher Anita Elberse, who studies the entertainment industry ( Elberse, 2013): “.. advances in digital technologies foster concentration and a winner-take-all dynamic (..). New technologies increasingly give people around the world access to the most sought-after television programs, movies, books, and opera performances.”

## Conclusions

Congresses will probably continue to be mostly onsite, with a more intensive integration of technology to reach members of the professional community who cannot attend the live events. As stated by a participant in a survey ( PCMA, 2020), “face-to-face will return, but with a need to better incorporate digital in what were previously face-to-face only events.”

The main stakeholders will continue to have an important role in the new state of affairs. However, those organizations that adapt more quickly will concentrate more of the share, as seen with the entertainment and the news industries ( Elberse, 2013; Lee and Molla, 2018). Many of them are already acting internationally. These actions can be propelled by the use of technology.

## Take Home Messages

• Meetings of the future will probably utilize technology more extensively to reach members of the professional community who cannot attend the live events.
• The Extended Congress concept is one of the possible formats to be used, extending a traditional meeting in the dimensions of time, space and languages.
• The current stakeholders will continue to have an important role in the new state of affairs.
• Those organizations which adapt more quickly to implement these new models will concentrate more of the share, as seen with the adoption of technology by the entertainment and news industries.

## Notes On Contributors

**Alvaro Margolis** is an internist with a Master’s degree in Medical Informatics from the University of Utah (USA). He has held academic positions at the Schools of Medicine and Engineering, Universidad de la República, Uruguay, is Founding Member of the International Academy of Health Sciences Informatics, and Associate Editor of Applied Clinical Informatics. He is the President of the Global Alliance for Medical Education (GAME), and Director of EviMed, a CME company working across the Americas. ORCID: https://orcid.org/0000-0002-2631-2323

**Jann Torrance Balmer** PhD RN serves as the Director for Continuing Medical Education (CME) of the University of Virginia School of Medicine and as the Co-Lead Nurse Planner for the School of Nursing. Dr. Balmer serves on the NPD Commission on Accreditation for the American Nurses Credentialing Center. She served on the American Board of Medical Specialties Vision Commission, and as the President, Past President and Board Member of the Alliance for Continuing Education in the Health Professions (formerly Alliance for CME) from 2005 - 2012.

**Andrea Zimmerman**, EdD is learning engineer at the Office of CME of the University of Virginia, and has worked in higher and continuing education since 2007 to provide innovative and engaging learning opportunities to clinicians and biomedical scientists, bridging the gap between bench and bedside. Dr. Zimmerman serves on the Excellence in Educational Design Award Committee for the Alliance for Continuing Education in the Health Professions. ORCID: https://orcid.org/0000-0002-3688-0842

**Antonio López-Arredondo** is a computer engineer, Adjunct Professor at the Health Informatics Laboratory, Universidad de la República, Uruguay. He is also Chief Technology Officer at EviMed.

## Appendices

Access to the pilot extended congress (in Spanish)

https://redemc.net/congresoextendido

User: MedEdPublish

Password: February2019

## Declarations

Alvaro Margolis and Antonio López-Arredondo are directors at EviMed, responsible for the pilot program described in the article. Jann Balmer and Andrea Zimmerman have nothing to disclose.
