# Supplementary material for: The Extended Congress: Reimagining scientific meetings after the COVID-19 pandemic
Source: MedEdPublish (2016). 2020 Jun 22;9:128. [Version 1] doi: 10.15694/mep.2020.000128.1 (PMC10702683; doi:10.15694/mep.2020.000128.1)
Supplement: Peer review history

# Reviewer report: Reviewer response for version 1

**DOI:** 10.21956/mep.20090.r30939

**Published:** 2020-07-20

**Authors:** Jocelyn Lockyer

**Affiliations:** 1 University of Calgary

This review has been migrated. The reviewer awarded 5 stars out of 5 The Margolis et al. (2020)1 publication should provide encouragement to organizations developing and implementing plans to continue their CPD work during and after this pandemic. There is no doubt that the CPD organizational survivors will be those who have determined ways to move into virtual and/or hybrid formats. The authors provide excellent and timely examples from their own work—the Latin American Peritoneal Dialysis Extended Congress and from the University of Virginia School of Medicine and Nursing. They offer practical advice on transitioning but also practical tips on how to make it happen and make it effective. They recognize the challenges of ensuring technological support, sufficient funding, marketing and online networking so that participants can engage with others as they would do at a face/face event.Hand-in-hand with the virtual conference is the need to support conference attendees to ensure they maximize their learning. A recent article by Tobin (2020)2 provides some guidance for learners. He presents several key strategies—going to a smaller program the first time, getting ready by blocking off the calendar so that the day is focused on learning, getting familiar with the software tools that will be available, taking notes and chatting with others during and after the session to consolidate ideas, be in the moment and realize you are unlikely to view on-line presentations later. For both the CPD organization and the learner, planning ahead and anticipating how to maximize the experience is key. It is a learning journey for everyone but worth the plunge. There is no doubt that learner expectations for safe high quality programming will be different post-COVID. 1. Margolis A, Balmer J, Zimmerman A, López-Arredondo A, 2020, 'The Extended Congress: Reimagining scientific meetings after the COVID-19 pandemic', MedEdPublish, 9, [1], 128, https://doi.org/10.15694/mep.2020.000128.12. Tobin TJ, How to make the most of a virtual conference, Chronical of Higher Education, July 14, 2020. https://www.chronicle.com/article/How-to-Make-the-Most-of-a/249171

Reviewer Expertise:

NA

No decision status is available

# Reviewer report: Reviewer response for version 1

**DOI:** 10.21956/mep.20090.r30942

**Published:** 2020-07-11

**Authors:** Samar Aboulsoud

**Affiliations:** 1 CAIRO UNIVERSITY, SCHOOL OF MEDICINE

This review has been migrated. The reviewer awarded 4 stars out of 5 I would like to thank the authors for presenting a topic that is so relevant and useful for the current situation. As we are all shifting to digital formats for education and online communication, it is definitely important to introduce and test new concepts that can support and improve the electronic modes of learning.The extended congress model has a great opportunity to be widely used in the world of virtual learning. However, and as the concept is still in its early days, it is essential to use a comprehensive evaluation model to evaluate the learning process and to analyze data and feedback from different stakeholders over a reasonable period of time. It will also be interesting to test the applicability of the extended congress model to a variety of settings e.g. different countries, cultures, and healthcare professions. If this model proves its effectivity, I believe it will provide lots of opportunities for inter-professional education. It will also be helpful to delineate the difference between “extended in space” and the traditional virtual conference. The expanded time is an interesting concept that can reinforce the learning but I believe this requires effective techniques that ensure the engagement of participants, which might be a challenge, especially over a long period of time.For the readers’ convenience, it would be nice to have a summary of the article. Thank you again for the opportunity to review this paper and I will definitely be interested to use the concept as an educational modality and I am really keen to read a follow up on this article and to learn more about the extended congress model as it ripens and matures.

Reviewer Expertise:

NA

No decision status is available

# Reviewer report: Reviewer response for version 1

**DOI:** 10.21956/mep.20090.r30940

**Published:** 2020-07-05

**Authors:** Angel Centeno

**Affiliations:** 1 Universidad Austral

This review has been migrated. The reviewer awarded 4 stars out of 5 Interesting paper on a timely problem: medical meetings during the pandemic.The authors present the example of one meeting that rapidly changed its format to the extended format. After an interesting introduction to the topic, they conclude that this is an option for the future.Would be interesting to have more information on aspects such as the continuity and engagement of the participants during the five weeks: does it decay?, is there any information concerning their active personal interaction? They mention “reasonable results” that need to be specified.Besides, if networking is one of the basic objectives of these meetings it is not clear how it can be sustained and promoted with this new format. A comment on that would be interesting.Are there any special characteristics of institutions that would benefit more (or less) of this format?In short it is a valid option for scientific meetings, and adding to the economic and financial aspects, the academic value of the extended meeting needs to be considered in detail.

Reviewer Expertise:

NA

No decision status is available

# Reviewer report: Reviewer response for version 1

**DOI:** 10.21956/mep.20090.r30941

**Published:** 2020-06-30

**Authors:** John Parboosingh

**Affiliations:** 1 University of Calgary, Canada

This review has been migrated. The reviewer awarded 5 stars out of 5 The authors describe an educational design that has the potential to foster group discussions leading to collective reflection and sequential activities over time, features that foster knowledge translation into practice. The article succeeds as intended, to start a dialogue with the medical education and events community about delivery formats with the potential to achieve this ambitious goal. While many readers including CPD providers will focus on the obvious challenges of adopting extended congresses, such as the costs and ethics and acceptance of new technologies, I use this review to highlight an equally important issue which needs to be included in this dialogue. The extended congress has the potential to provide a home (1) for health professionals to regularly network with peers and engage in self-managed learning activities that inform practice. This goal is best achieved by changing how we think about learning and planning new programs. For instance, we should think of learning as emerging from continuing conversations among networked participants who, owning the education process, create content relevant to current practice and ‘pull’ evidence-based best practices into the conversation. (2) Relationships among trusted peers provide practitioners with the motivation to talk about their practice, critically reconstructing practice incidents as they do informally at work, for instance at coffee and lunch breaks. (3) Such engagement should be a prime objective in extended congresses. As Anand states describing HBX, the Harvard Business School’s learning platform, “If we could crack the code of engagement, we felt, reach would follow”. (4)For extended congresses to achieve these goals there is a need for planning and support teams to include information and network specialists, as mentioned by the authors, but also professionals with expertise in social learning (5) and community facilitation (6).1. Graham T. McMahon (2016) What Do I Need to Learn Today? — The Evolution of CME, N ENGL J MED 374;15.2. Jordan ME, Lanham HJ, Crabtree BF, et al. The role of conversation in health care interventions: enabling sensemaking and learning. Implement Sci. 2009;4:15.3. Lanham HJ, McDaniel RR Jr, Crabtree BF, et al. How improving practice relationships among clinicians and non-clinicians can improve quality in primary care. J Comm J Qual Patient Saf. 2009;35:457–466.4. Anand B. (2016). Chapter 29. The Content Trap: A Strategist's Guide To Digital Change, Random House.5. FutureLearn’s Social Lead, David Thair, shares his top six tips for getting the most out of social learning features https://about.futurelearn.com/blog/6-social-learning-tips6. Jakubec S, Parboosingh J, Colvin B. Introducing a multimedia course to enhance health professionals’ skills to facilitate communities of practice: experiences of the first cohort of course participants. J Health Organ Manage. 2014;28(4):477–494.

Reviewer Expertise:

NA

No decision status is available

# Reviewer report: Reviewer response for version 1

**DOI:** 10.21956/mep.20090.r30943

**Published:** 2020-06-25

**Authors:** Helena Filipe

**Affiliations:** 1 Hospital of the Armed Forces/PL-EMGFA

This review has been migrated. The reviewer awarded 5 stars out of 5 Thank you for the opportunity to review the manuscript, which I read with great pleasure.It reads well and despite long, the reader is urged to read along with continuing interest.If already relevant, the pandemic made central, the topic approached by Margolis and Associates. The way Authors introduce their writing purpose: a “starting dialogue” with the reader and the whole CPD community and the innovative learning methodology they introduce and propose: the “Extended Congress” raise immediate and unwavering curiosity till the conclusion of the article.The Authors, two at least are renowned experienced educators, start by comprehensive and systematically framing their work in evidence. They picture the difference between the model they propose with previous or ongoing others and underline the benefit of theirs and why they developed it. A table could be built upon this.The “Extended Congress” model allows for a triad learning expansion (space, time and languages), which Authors very elegantly sustain. After wisely balancing the support of their model on evidence and prior experience, they provide a clear description of its implementation. A note of remark for caring to develop a blended model offering learning experiences encompassing dialogue and conversation beyond discussion, self and collective reflection on new learning and, including an online networking space to develop relationships and further new learning. The methodology is clearly explained to the point of replication and embraces social learning principles, active learning, multifaceted formats and incorporates the longitudinal component. All of those have been associated to effective CPD programs. Thank you for itemizing the challenges encountered and overcome.The Authors explained the importance of establishing bonds and partnerships to create and implement these kinds of CPD programs. The article provides a holistic perspective on longitudinal hybrid CPD programs and that includes all stakeholders usually involved in CPD live programs, challenges they can face and benefits these learning formats can bring. Probably new actors will emerge, some probably evolving from reshaping preexisting roles. New and exciting challenges are posed to CPD educators. Interesting thought on progressing the model eventually considering virtual reality. Would probably include this section as a possible line of continued investigation. There is a good discussion section and the expression“refocus energy to something you’ve already built rather than scramble to reinvent the wheel." translates well the authors´ vast experience and previous work on this medical education area. The take home messages were surgically extracted and summarize well the whole article.The supplementary file is an excellent standalone document showing a comprehensive and useful checklist to eventually underpin a future rubric to guide organizing teams to plan and evaluate learning experiences. Looking forward to reading the next article expanding on the impact of the “Extended Congress” model on several evaluation levels and to learn about further developments.References: RM Cervero, JK. Gaines, The Impact of CME on Physician Performance and Patient Health Outcomes: An Updated Synthesis of Systematic Reviews Journal of Continuing Education in the Health Professions, 35(2):131–138, 2015IJ Parboosingh, VA. Reed, JC Palmer, HH Berstein. Enhancing Practice Improvement by Facilitating Practitioner Interactivity: New Roles for Providers of Continuing Medical Education Journal of Continuing Education in the Health Professions, 31(2):122–127, 2011

Reviewer Expertise:

NA

No decision status is available
